# Pre- and postoperative 4D CT analysis of thumb CMC joint kinematics after first metacarpal extension osteotomy

**DOI:** 10.1007/s00402-026-06455-5

**Published:** 2026-08-27

**Authors:** Alissa Guebeli, Philipp Honigmann, Johannes G. G. Dobbe, Marco Keller

**Affiliations:** 1 Hand and Peripheral Nerve Surgery, Department of Orthopaedic and Trauma Surgery, Kantonsspital Baselland, Liestal, Switzerland; 2 The Hand Clinic - Geneva, Geneva, Switzerland; 3 Hand Center Northwestern Switzerland, Pratteln, Switzerland; 4 Medical Additive Manufacturing Research Group (MAM), Department of Biomedical Engineering, University of Basel, Allschwil, Switzerland; 5 Department of Biomedical Engineering and Physics, location AMC, Amsterdam University Medical Center, Amsterdam, Netherlands; 6 Amsterdam Movement Sciences, Musculoskeletal Health—Restoration and Development, location AMC, Amsterdam University Medical Center, Amsterdam, Netherlands; 7 Department of Orthopaedic Surgery, Traumatology and Hand Surgery, Spital Limmattal, Schlieren, Switzerland

**Keywords:** Carpometacarpal joint, Thumb, Four-dimensional computed tomography, Wilson osteotomy

## Abstract

**Introduction:**

First metacarpal extension (Wilson) osteotomy combined with modified Brunelli ligamentoplasty is performed for early thumb carpometacarpal (CMC1) osteoarthritis to improve pain and function. The purpose of this study was to quantify in vivo changes in CMC1 joint contact distribution and kinematics after this combined procedure and to determine whether postoperative patterns resemble those of healthy volunteers. We hypothesized that the procedure would reduce cumulative palmar contact during opposition–retroposition.

**Materials and methods:**

In a prospective single-center observational cohort study, six adults with early CMC1 osteoarthritis (Eaton–Littler I–II) underwent standardized preoperative and 6-month postoperative four-dimensional computed tomography (4D CT) of the CMC1 joint during active opposition–retroposition. Five healthy volunteers served as reference with a single 4D CT scan. First metacarpal–trapezium motion was registered across frames. Joint contact was estimated using a 2.0 mm proximity surrogate on the trapezial surface. The primary outcome was change in cumulative palmar contact area. Secondary outcomes included change in dorsoradial contact, temporal variability of contact curves, and contact-centroid path length and mean position. Within-subject pre- versus postoperative comparisons were performed; healthy scans provided reference patterns.

**Results:**

Healthy volunteers showed reproducible patterns with increasing palmar contact toward terminal opposition and near-mirror symmetry on retroposition. Preoperatively, patients demonstrated elevated palmar contact persisting into retroposition and loss of symmetry. Postoperatively, the procedure did not consistently reduce cumulative palmar contact. Instead, most patients exhibited increased palmar contact, flattening of contact-versus-time curves, and a shorter contact-centroid path with a palmar–ulnar shift in mean position. These changes suggest a stabilizing effect more consistent with functional partial arthrodesis than selective palmar unloading.

**Conclusion:**

Wilson osteotomy with modified Brunelli ligamentoplasty did not produce the anticipated palmar unloading during functional thumb motion. The dominant postoperative effect was palmar loading with reduced dynamic variability. These findings refine the biomechanical rationale for this joint-preserving procedure and support future studies isolating the osteotomy from ligamentoplasty using load-standardized tasks.

**Level of evidence:**

Therapeutic, IV.

**Supplementary Information:**

The online version contains supplementary material available at https://doi.org/10.1007/s00402-026-06455-5.

## Introduction

 Thumb carpometacarpal (CMC1) osteoarthritis impairs pinch, grip, and dexterity. Extension, or closing-wedge osteotomy of the first metacarpal (Wilson osteotomy) is a viable, joint-preserving option for early-stage disease after failure of nonoperative treatment [1, 2]. By resecting a 20–30° dorsal closing wedge at the metacarpal base, the procedure aims to redistribute contact and load from the volar aspect of the first CMC joint to healthier dorsal regions and to improve joint stability. Long-term follow-ups generally report significant pain relief and functional improvement, with variable effects on strength in younger patients [3–8].

Patient-specific planning and guides have improved correction reliability for first metacarpal (MC1) osteotomy, and contemporary 3D axis analyses suggest typical correction magnitudes in the low-to-mid 20° range when planned approximately 1–1.5 cm distal to the joint line [9, 10]. In many practices, osteotomy is combined with a CMC1 ligamentoplasty to address both load redistribution and joint stabilization [11, 12].

Despite favorable clinical outcomes and a persuasive cadaveric foundation, the in-vivo biomechanical effect of extension osteotomy during functional motion has not been confirmed. Pellegrini and colleagues demonstrated in cadavers that extension osteotomy can unload palmar contact areas and shift loading dorsally in normal and moderately arthritic joints using pressure-sensitive film [13].

Four-dimensional computed tomography (4D CT) now enables motion-resolved imaging of the CMC1 joint. Early clinical work established feasibility during circumduction, followed by in vivo studies in healthy volunteers that characterized opposition/abduction kinematics and estimated articular contact patterns, and cadaveric dynamic CT studies that quantified opposition with high interspecimen consistency and low radiation dose [14–16]. More recently, post-intervention dynamic imaging has captured biomechanical changes after dorsoradial ligament reconstruction in symptomatic, pre-arthritic CMC instability, highlighting the sensitivity of dynamic CT to surgical effects [17]. Task-standardized, force-controlled 4D CT during dynamic pinch has further shown force-dependent rotations and translations of the first metacarpal relative to the trapezium in vivo [18]. Methodological advances—including automated and semi-automated analysis pipelines—now standardize kinematic metrics, hysteresis, and proximity-based contact estimation across the motion cycle and facilitate larger cohort analyses, while patient-specific planning and guides improve correction fidelity for first-metacarpal osteotomy, and 3D axis analyses help target typical correction magnitudes in the low-to-mid 20° range [9, 10, 19, 20]. Recent work using 3D CT–based analysis reported small, nonsignificant dorsal–radial shifts of the peak load zone and a tendency toward flatter pressure distributions after basal osteotomy. This underscores the value of standardized, image-based surrogates for contact while highlighting the need for in vivo dynamic assessment [21].

Emerging biomechanical work using CT-based subchondral bone density mapping, complemented by arthroscopy, suggests postoperative stress normalization and potential cartilage repair after extension–abduction osteotomy, underscoring the need to reconcile clinical benefits with in vivo mechanics [22].

Despite these advances, no prospective in vivo study has directly tested whether first metacarpal extension osteotomy achieves the intended palmar unloading during functional thumb motion. We therefore conducted a prospective observational study using pre- and postoperative 4D CT to analyze CMC1 contact distribution and kinematics during opposition–retroposition, and we compared postoperative patterns with healthy volunteer references.

## Materials and methods

A prospective analysis was performed on patients planned for combined Wilson osteotomy and modified Brunelli ligamentoplasty for CMC1 joint osteoarthritis in our unit. We designed and reported the study according to the Strengthening the Reporting of Observational Studies in Epidemiology (STROBE) statement for cohort studies [23]. Ethical approval for the acquisition and use of the image data was granted by the regional ethics committee. To be able to evaluate and quantify the CMC1 contact surface area, all wrists in this study were CT scanned and the MC1 and trapezium were subsequently segmented. Motion of the thumb was acquired while conducting a 4D CT scan. Registration of the segmented MC1 and trapezium bones to all time frames of the scan enabled quantifying the entire motion trajectory and evaluating the surface area where the bones were in contact in each individual time frame. An anatomical coordinate system was determined for each trapezium and allowed showing where in the *xy*-plane the centroid of the contact surface was located during the motion trajectory. It further enabled subdividing the contact surface into four standardized quadrants of this *xy*-plane, and to evaluate variation of the surface area as a whole and in these quadrants.

### Patient group and scanning

In this study, six patients were included who underwent combined Wilson osteotomy and modified Brunelli ligamentoplasty. All patients were female, with a mean age of 44.5 years. Five healthy volunteers (two male, three female; mean age 35.8 years) served as controls for all evaluation experiments. Patient inclusion and study-associated interventions (Wilson osteotomy and 4D CT exams) were conducted between April 2021 and December 2022. All image data used in this study were obtained with written informed consent for use.

A spiral CT scan was made of all wrists for segmentation of the trapezium and MC1 bone. A single 4D CT scan was performed on healthy volunteers while they executed a 5 s opposition and 5 s retroposition motion of the thumb. For the patient group, similar 4D CT scans were conducted preoperatively and 6 months postoperatively, since the MC1 morphology had changed. All scans were made using a Siemens Somatom Force CT scanner (Siemens, Erlangen, Germany). Each 4D CT scan contained 33 timeframes for opposition and 33 for retroposition of the thumb. To be able to compare all quantitative results, all scans of left hands were mirrored.

The standard settings for the spiral 3D CT scans were 120 kV, 65 mAs, slice thickness 0.6 mm, convolution kernel Br59s\3. The settings for the nonspiral 4D CT scans were 120 kV, 65 mAs, slice thickness 0.6 mm, convolution kernel Br59\3, gantry rotation time 0.25 s, 33 volume reconstructions per 4D CT scan. Image data were reconstructed into volume images with voxel spacing 0.3 × 0.3 × 0.3 mm.

### Motion analysis

Motion analysis was performed using custom software [24]. The MC1 and trapezium were segmented from the spiral CT scan using a Laplacian level-set segmentation growth algorithm, initialized by the result of threshold-connected region growing and subsequent filling of remaining gaps and closing of the outline. Intensity-based registration was used by subsampling the original CT image at ± 0.3 mm perpendicular to the surface of each bone model, hereby selecting bright points in the cortical bone layer, and dark points in surrounding soft tissue. The selection of bright and dark points rendered the registration procedure more discriminative when finding the bone objects in each of the time frames of the 4D CT scans. The registration procedure was semiautomatic, in which the user roughly aligns the bones manually with the first frame of the 4D CT image. Additionally, a downhill simplex optimization algorithm by Nelder and Mead was used to optimize positioning automatically in which the correlation coefficient between the source and target gray-level intensities served as optimization metric [25, 26]. Automatic registration to subsequent 4D CT frames continued from the previous frame, limiting further user interaction. Registration to each 4D CT frame provided two 4 × 4 matrices for aligning MC1 (**M**_mc_) and the trapezium (**M**_t_) with that target image. Relative positioning of MC1 with respect to the trapezium is therefore described by $\:\boldsymbol{M}\left(i\right)={\boldsymbol{M}}_{mc}^{-1}\left(i\right){\boldsymbol{M}}_{t}\left(i\right)$, with ***M***(*i*) the relative positioning matrix for time frame * i*.

### Finding a local coordinate system for each trapezium

**Fig. 1:**
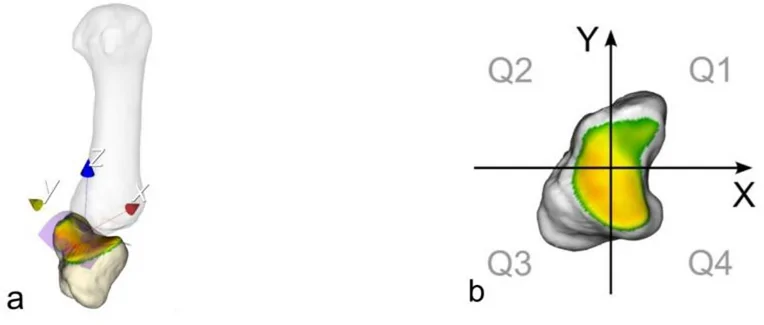
**a** Articular coordinate system and contact surfaces. The minimum dynamic distance map over the entire motion trajectory is clipped (colored surface), and a saddle function is fit (purple) to that surface providing the origin and orientation of the axes of the local coordinate system for one reference trapezium. The reference trapezium and coordinate system are aligned with all other trapeziums to define a local coordinate system for all trapeziums in the same way; **b** The contact surface area is clipped for each frame using the *xz* and *yz* planes of the local coordinate system to obtain the fraction of the surface area in each of the four quadrants (Q1, Q2, Q3, Q4) of the *xy*-plane

In this study the goal is to investigate the contact surface area in healthy individuals and in patients, before and after corrective osteotomy surgery, to project these surfaces in the *xy*-plane of a 3D coordinate system (CS), and to subdivide these areas over the quadrants of this *xy*-plane. It enables us to evaluate how the contact surface varies as a whole and in these four quadrants. This requires defining a coordinate system that sits roughly in the middle of the contact surface of a full motion cycle in a healthy individual. To this end a trapezium out of the healthy set with median volume was chosen as reference. The minimum distance map over the entire motion trajectory is determined and the surface within 0–2 mm from the MC1 bone is clipped from the trapezium. This contact surface is shaped like a saddle and is therefore fit with a saddle function, hereby yielding a 3D anatomical CS that fits the contact surface as good as possible (Fig. 1a). The axes are in the following anatomical directions: +*x* in dorsal direction of the thumb, +*z* pointing towards MC1 and + *y* perpendicular to the + *x* and + *z* axes according to the right-hand rule.

In this study we aim to compare the frame-by-frame contact surfaces of MC1 with the trapezium, as equally as possible between individuals. We therefore used Iterative Closest Point registration to align the reference trapezium –and its anatomical CS- to the remaining trapeziums, to define a local CS for all these trapeziums in the same way [27]. The contact surface area is projected into the quadrants of the *xy*-plane as depicted in Fig. 1b.

### Contact surfaces

Contact surfaces were determined for each frame in the 4D CT scans by applying the relative positioning matrix ***M***(*i*) to MC1, to bring it in the right relative position with respect to the trapezium. With the bones in such pose, the nearest neighbor distance was determined for each mesh point of the trapezium to the point cloud of the MC1 mesh. Subsequently, the surface area was clipped out of the trapezium for which the distance value was in the range [0, 2] mm. This procedure was repeated for relative position of MC1 and the trapezium in all 4D CT frames, yielding 66 surface areas for the trapezium, representing the contact area for the entire opposition and retroposition trajectory. Each contact surface area was clipped using the *xz*- and *yz*-planes of the local coordinate system to obtain the fraction of the surface area in each of the four quadrants of the *xy*-plane. In addition, the centroid of the contact surface was projected onto the *xy*-plane of the local coordinate system to define its projected location.

### Statistical analysis

Continuous variables are presented as median (interquartile range). For palmar contact surface area (sum of quadrants 2 and 3), the difference between pre- and postoperative measurements within the patient group was assessed with the Wilcoxon signed-rank test, while comparisons between healthy volunteers and preoperative patients and between healthy volunteers and postoperative patients were performed with the Mann–Whitney U test. For centroid path length, an overall difference among the three groups was evaluated with the Kruskal–Wallis test, followed by pairwise Mann–Whitney U tests. Effect sizes are reported as rank-biserial correlation for the Wilcoxon and Mann–Whitney tests and as ε² for the Kruskal–Wallis test. Contact surface area in the individual quadrants was summarized descriptively for each group. All tests were two-sided and a p-value < 0.05 was considered statistically significant. Statistical analyses were performed with jamovi (version 2.7.31.0).

## Results

### Cohorts and data quality

Five healthy volunteers (H1–H5) and six patients (P1–P6) completed analyzable motion cycles (opposition - retroposition) during 4D CT. Example 4D CT videos of one healthy volunteer (H2) and one patient (P3), including preoperative and postoperative scans, are provided in Online Resources 1–3. All left hands were mirrored into a right-hand coordinate system. Proximity mapping yielded time-resolved contact estimates over the trapezial surface subdivided into four quadrants: Q1 (dorsal–ulnar), Q2 (palmar–ulnar), Q3 (palmar–radial), and Q4 (dorsal–radial). Contact-centroid coordinates (*x*, *y*) were used for path-length and mean position analyses (negative *x* = palmar; positive *y* = ulnar) (Fig. 1b).

### Healthy volunteer reference patterns

**Fig. 2:**
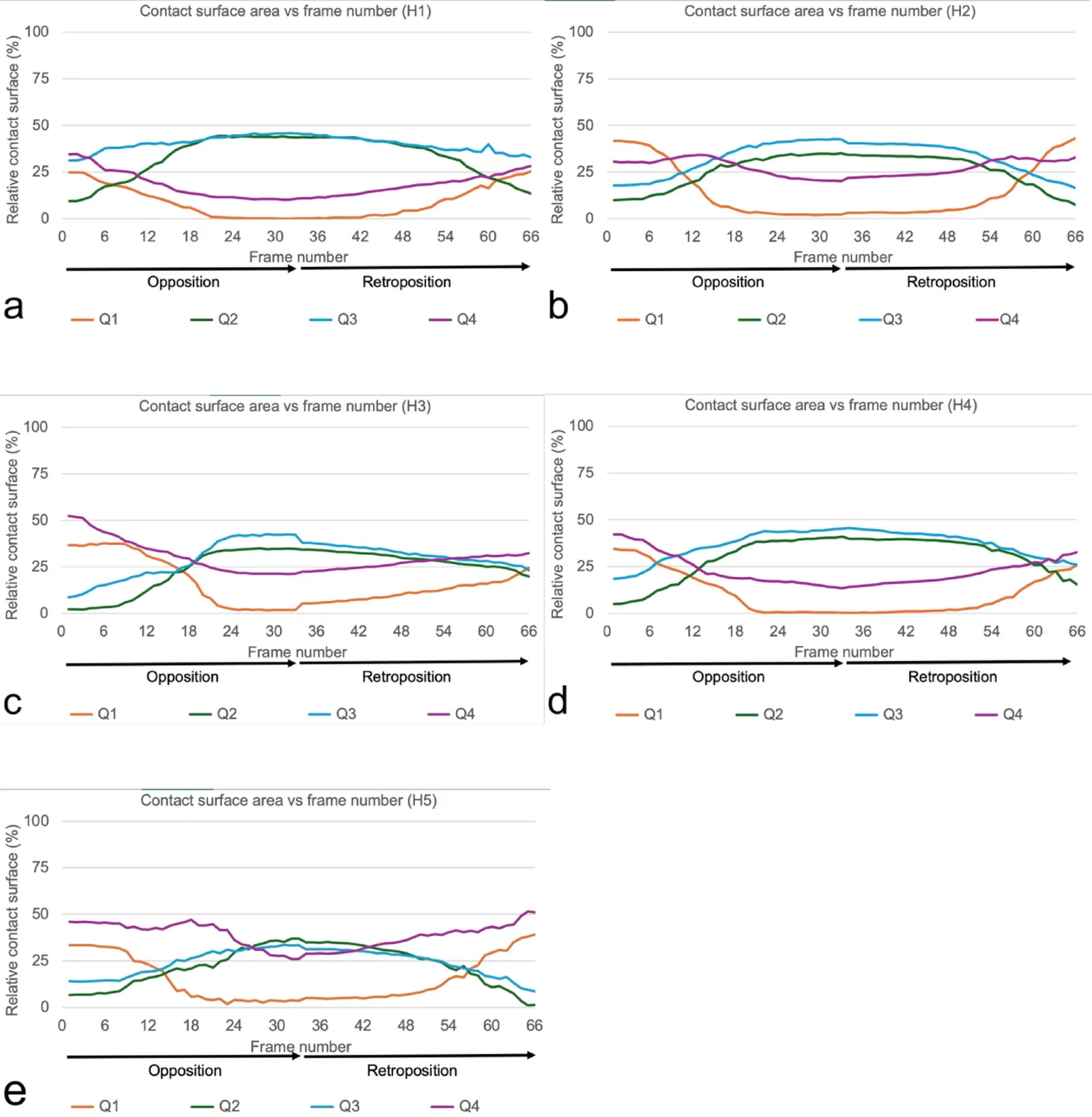
**a–e** Articular contact surface area during motion in healthy subjects. The figures visualize the contact surface percentage in each of the four quadrants at different time points during opposition – retroposition motion of healthy subjects H1-H5

Across healthy volunteers, contact-versus-frame number curves were highly reproducible, supporting methodological validity and good task standardization despite the absence of a powered guidance device (Fig. 2a–e). Table 1 summarizes the average contact surface area of each quadrant during opposition and retroposition.

**Table 1:** Average surface contact area (mm^2^; %) of each quadrant (Q1-Q4) for healthy individuals (H1-H5) during opposition and retroposition

Movement	#	Q1	Q2	Q3	Q4
Opposition	H1	10.3; 8.8	40; 32.3	50; 40.8	21.7; 18.1
Opposition	H2	19.8; 15.5	29.9; 24.8	38.7; 32	33.9; 27.6
Opposition	H3	21.1; 19.7	22.5; 20.6	30.3; 27.8	34.3; 31.9
Opposition	H4	12.7; 13.1	26.8; 27.1	35.1; 35.7	23.2; 24
Opposition	H5	12.7; 11.9	35.9; 32.3	34.1; 30.9	27.2; 25
	Mean	15.3; 13.8	31; 27.4	37.6; 33.4	28.1; 25.3
	SD	4.8; 4.1	7; 5	7.5; 5	5.9; 5.1
Retroposition	H1	10; 8.5	42.6; 34	48.9; 39.4	22; 18
Retroposition	H2	16.4; 13.1	32.5; 26.7	40.2; 33	33.4; 27.2
Retroposition	H3	13.1; 11.8	32.3; 29	35.5; 31.8	30.5; 27.4
Retroposition	H4	7; 7.1	32.9; 33.8	36.9; 37.8	20.9; 21.3
Retroposition	H5	15.7; 14.8	27.1; 23.7	27.3; 24	41.3; 37.5
	Mean	12.5; 11.1	33.5; 29.4	37.7; 33.2	29.6; 26.3
	SD	3.9; 3.2	5.6; 4.5	7.8; 6	8.4; 7.4

^#^ Participant identification

Typical features included:

Palmar quadrants (Q2/Q3): contact surface area was highest in Q3 during both opposition (mean 33.4%) and retroposition (mean 33.2%). Cumulative contact increased toward the end of opposition; Q3 (palmar–radial) generally started slightly higher than Q2 (palmar–ulnar), and the curves converged near terminal opposition—consistent with the known pronation/adduction component of opposition [28].

Dorsal quadrants (Q1/Q4): Q4 (dorsal–radial) was consistently higher than Q1 (dorsal–ulnar) at motion start, indicating greater radial contact in the starting position; through opposition, Q1 and Q4 tended not to converge and frequently diverged further, leaving the dorsal joint radially “tighter” at maximal opposition.

Retroposition: typically mirrored opposition, with contact returning to baseline patterns.

### Patients’ preoperative patterns

During opposition, preoperative patient curves resembled healthy patterns. In retroposition, however, there was a shift toward higher Q3 (mean 39.4%) and lower Q1 (mean 6.54%) (Table 2). Palmar quadrants, especially Q2/Q3, remained relatively “tight” and did not fall back to their initial level by cycle end in some cases (unlike healthy scans) (Fig. 3a, c, e, g, i, k). Statistically, no significant difference in palmar contact surface area was observed between preoperative patients and healthy volunteers (median 63.2% [IQR 12.6] versus 58.3% [IQR 11.8]; Mann–Whitney U = 13.0, *p* = 0.792, rank-biserial correlation = − 0.133) (Table 3).

Fig. 3**a–l** Articular contact surface area during motion in patients. The figures illustrate the relative contact surface area within each of the four joint quadrants at sequential time points during opposition–retroposition motion in patients P1–P6. Preoperative measurements are shown in panels **a**, **c**, **e**, **g**, **i**, and **k**; postoperative measurements in panels **b**, **d**,** f**, **h**, **j**, and **l**

**Figure:**
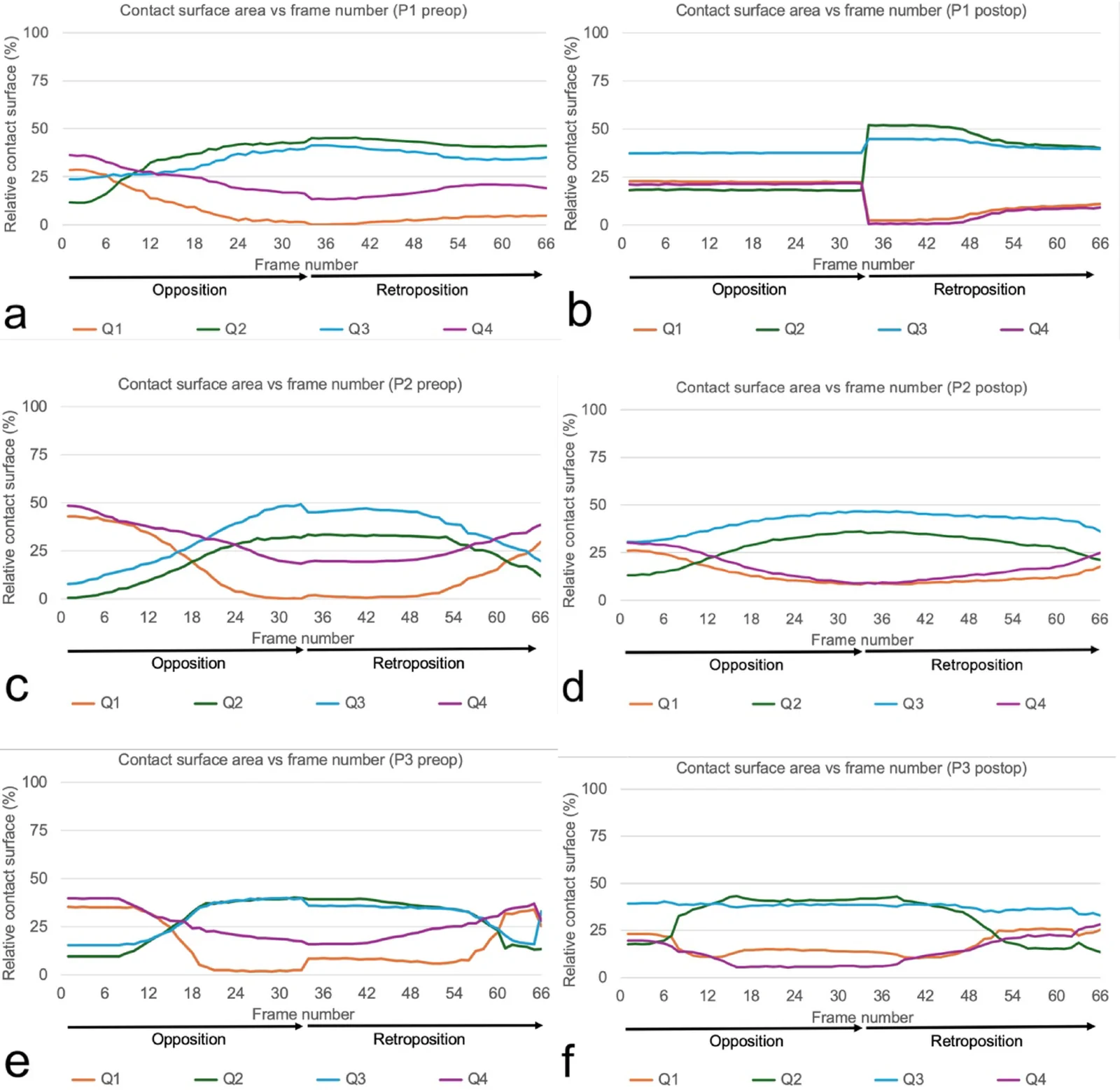

**Figure:**
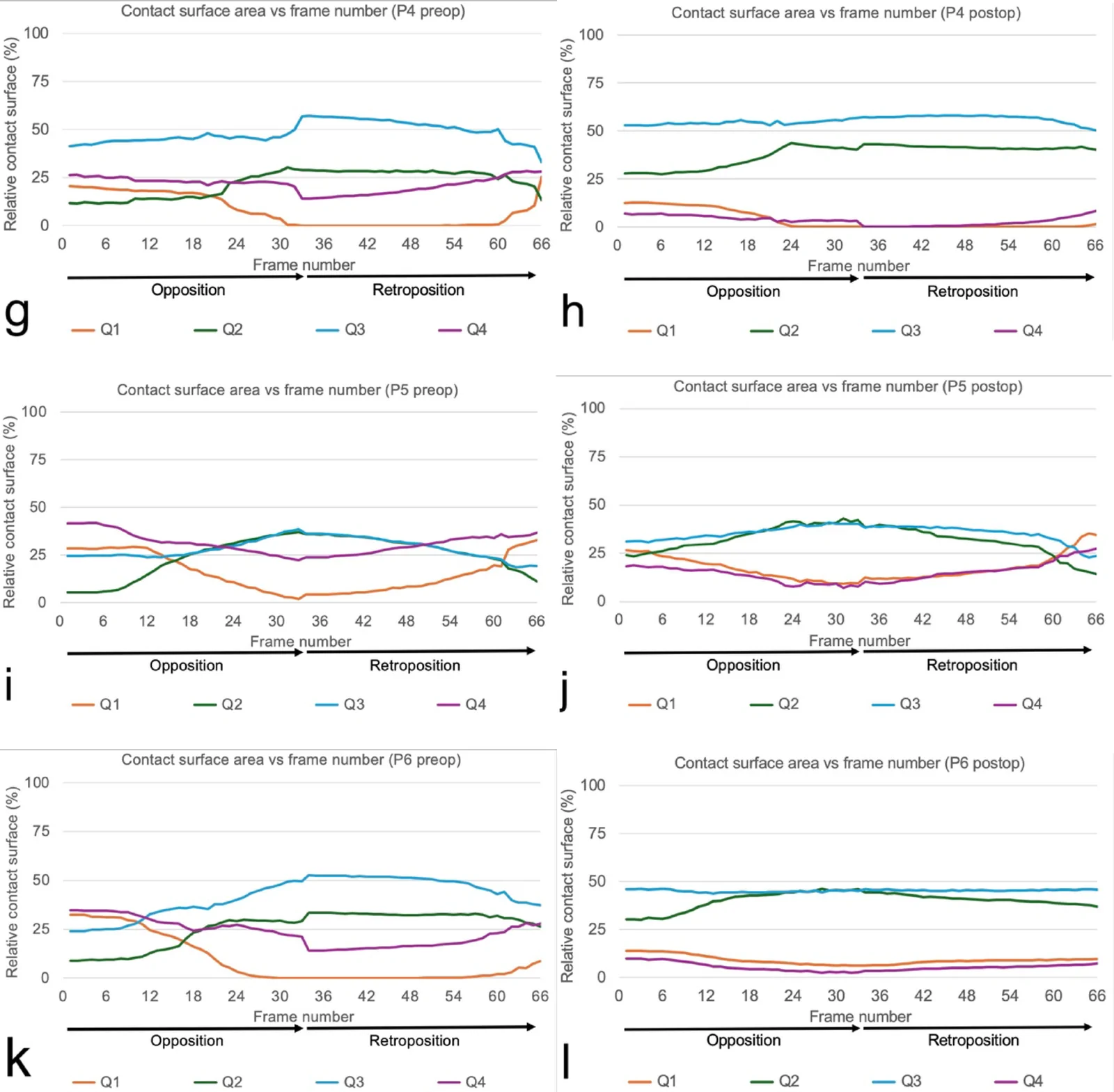


**Table 2:** Average surface contact area (mm^2^; %) of each quadrant (Q1-Q4) for patients (P1-P6) during opposition and retroposition before (PREOP) and after (POSTOP) Wilson osteotomy and Brunelli ligamentoplasty

Movement	#	Q1	Q2	Q3	Q4
PREOP					
Opposition	P1	12.7; 11.9	35.9; 32.3	34.1; 30.9	27.2; 25
Opposition	P2	22.2; 21.4	17.5; 17.3	28.2; 27.7	34.5; 33.6
Opposition	P3	24.8; 17.6	35.3; 26.2	37.6; 27.9	38.8; 28.2
Opposition	P4	12.2; 14	16.3; 17.5	40.7; 45	21; 23.4
Opposition	P5	21.1; 18.4	25.6; 21.4	32.7; 28	37.2; 32.2
Opposition	P6	17.1; 16.5	20.8; 19.7	37.5; 35.5	29.8; 28.4
	Mean	18.3; 16.7	25.2; 22.4	35.2; 32.5	31.4; 28.5
	SD	5.2; 3.4	8.7; 5.9	4.4; 6.8	6.7; 4
Retroposition	P1	3.1; 2.7	48.8; 42.7	42.5; 37.2	19.9; 17.4
Retroposition	P2	7.8; 7.7	29.4; 28.6	40.3; 39.2	25.2; 24.6
Retroposition	P3	18; 13.1	43.9; 31.8	43.2; 31.5	32.2; 23.6
Retroposition	P4	2; 1.9	28.3; 26.7	54; 51.1	21.4; 20.3
Retroposition	P5	15.7; 12.6	34.6; 28.3	35.5; 29.1	36.9; 30
Retroposition	P6	1.3; 1.2	37.4; 32	56.6; 48.4	21.3; 18.5
	Mean	8; 6.5	37.1; 31.7	45.4; 39.4	26.2; 22.4
	SD	7.3; 5.4	8.1; 5.8	8.2; 8.9	6.9; 4.7
POSTOP					
Opposition	P1	(*)	(*)	(*)	(*)
Opposition	P2	15.5; 15.6	26.4; 25.9	40; 39.4	19; 19.1
Opposition	P3	19; 15.6	43.4; 35.7	47.4; 38.8	12.4; 10
Opposition	P4	5.4; 6.6	31.4; 34.5	48.5; 54.2	4; 4.7
Opposition	P5	15.6; 16.8	30.8; 34	32.7; 35.9	12.3; 13.3
Opposition	P6	10.3; 9.7	41.9; 39.7	47.7; 45	6.1; 5.6
	Mean	13.2; 12.9	34.8; 33.9	43.3; 42.7	10.8; 10.5
	SD	5.3; 4.5	7.4; 5	6.8; 7.3	5.9; 5.9
Retroposition	P1	7; 6.5	47.7; 46.4	43.8; 42.4	5.2; 4.7
Retroposition	P2	11.5; 10.9	32.7; 31	46; 43.7	15.1; 14.4
Retroposition	P3	23.4; 18.7	36; 27.7	47; 36.8	20.9; 16.7
Retroposition	P4	0.1; 0.1	42.5; 41.5	57.9;56.5	2.1; 1.9
Retroposition	P5	17.2; 17.8	29.7; 30.4	34.4; 35.3	15.9; 16.4
Retroposition	P6	9.7; 8.4	46.8; 40.9	52.2; 45.5	6; 5.2
	Mean	11.5; 10.4	39.2; 36.3	46.9; 43.4	10.9; 9.9
	SD	8.1; 7.1	7.5; 7.6	7.9; 7.6	7.5; 6.7

# Participant identification

(*) Patient did not move during postop opposition 4DCT scan

### Patients’ postoperative changes: primary analysis

Contrary to the a priori hypothesis of palmar unloading, postoperative scans did not demonstrate a consistent reduction in cumulative palmar contact (Q2/Q3). Instead, the dominant and most consistent change was a decrease in dorsal–radial (Q4) contact to approximately 10% together with a significant increase in palmar contact from a median 63.2% (IQR 12.6) preoperatively to 77.8% (IQR 18.4) postoperatively (Wilcoxon signed-rank test, W = 0.00, *p* = 0.031; rank-biserial correlation = − 1.00). The median difference was 15.9% (IQR 3.78). Postoperative palmar contact surface area was also significantly higher than in healthy volunteers (median 77.8% [IQR 18.4] versus 58.3% [IQR 11.8]; Mann–Whitney U = 3.00, *p* = 0.030, rank-biserial correlation = − 0.800) (Table 3; Fig. 3b, d, f, h, j, l).

**Table 3:** Cumulative articular contact area of palmar quadrants

Opposition					Retroposition			Combined motion		
Patient group			Healthy group		Patient group		Healthy group	Patient group		Healthy group
	Preop	Postop			Preop	Postop		Preop	Postop	
P1	63.1	(*)	H1	73	79.9	88.8	73.4	71.5	88.8	73.2
P2	45	65.3	H2	56.9	67.7	74.7	59.7	56.3	70	58.3
P3	54.1	74.5	H3	48.4	63.3	64.6	60.8	58.7	69.5	54.6
P4	62.5	88.7	H4	62.8	77.8	98	71.6	70.2	93.4	67.2
P5	49.4	69.9	H5	63.1	57.4	65.7	47.7	53.4	67.8	55.4
P6	55.2	84.7	-	-	80.4	86.4	-	67.8	85.5	-
Mean	54.9	74.6		60.9	71.1	79.7	62.6	62	77.9	61.7
SD	7.2	10.1		9.1	9.7	13.5	10.4	8.3	12.2	8.1

The table shows mean articular contact area in % of quadrants Q2 and Q3 during opposition, retroposition and combined of each patient (P1-P6) and healthy volunteer (H1-H5)

(*) Patient did not move during postop opposition 4D CT scan

### Patients’ postoperative changes: secondary analyses

Temporal variability: contact curves flattened in several patients, reflecting reduced rise in palmar contact during opposition and a more uniform profile across the motion cycle with reduced cyclic peaks. This pattern supports a stabilizing effect on dynamic contact.

Centroid kinematics: the contact-centroid path length decreased postoperatively, indicating reduced excursion of the contact locus across the joint surface, which we interpret as a functional partial arthrodesis behavior. The mean centroid position shifted palmar–ulnar, in the opposite direction of the intended dorsal–radial correction vector (Fig. 4a–l). Statistically however, there was no overall difference in centroid path lengths among healthy volunteers, preoperative patients and postoperative patients (Kruskal–Wallis χ²(2) = 4.53, *p* = 0.104, ε² = 0.302). Healthy volunteers and preoperative patients showed comparable values (median 8.16 mm [IQR 1.11] versus 7.19 mm [IQR 1.54]; Mann–Whitney U = 8.00, *p* = 0.247, rank-biserial correlation = − 0.467). Postoperative patients had a shorter centroid path length than healthy volunteers (median 5.61 mm [IQR 4.18] versus 8.16 mm [IQR 1.11]; Mann–Whitney U = 3.00, *p* = 0.056, rank-biserial correlation = 0.760). The difference between pre-operative and post-operative patients was not significant (median 7.19 mm [IQR 1.54] versus 5.61 mm [IQR 4.18]; Mann–Whitney U = 9.00, *p* = 0.329, rank-biserial correlation = − 0.400). Although the post-operative reduction relative to healthy controls did not reach conventional statistical significance, the large effect size indicates a potentially relevant shortening of the centroid path after Wilson osteotomy (Table 4).

Fig. 4**a–l** Centroid position during motion in patients. The figures illustrate the position and path of the articular contact centroid during opposition–retroposition motion in patients P1–P6. Preoperative centroid trajectories are shown in panels **a**, **c**, **e**, **g**, **l**, and **k**; postoperative trajectories in panels **b**, **d**, **f**, **h**, **j**, and **l**. The *x*-axis represents the palmar–dorsal direction, and the *y*-axis represents the radial–ulnar direction. Positive *x* values indicate dorsal position, and positive *y* values indicate ulnar position

**Figure:**
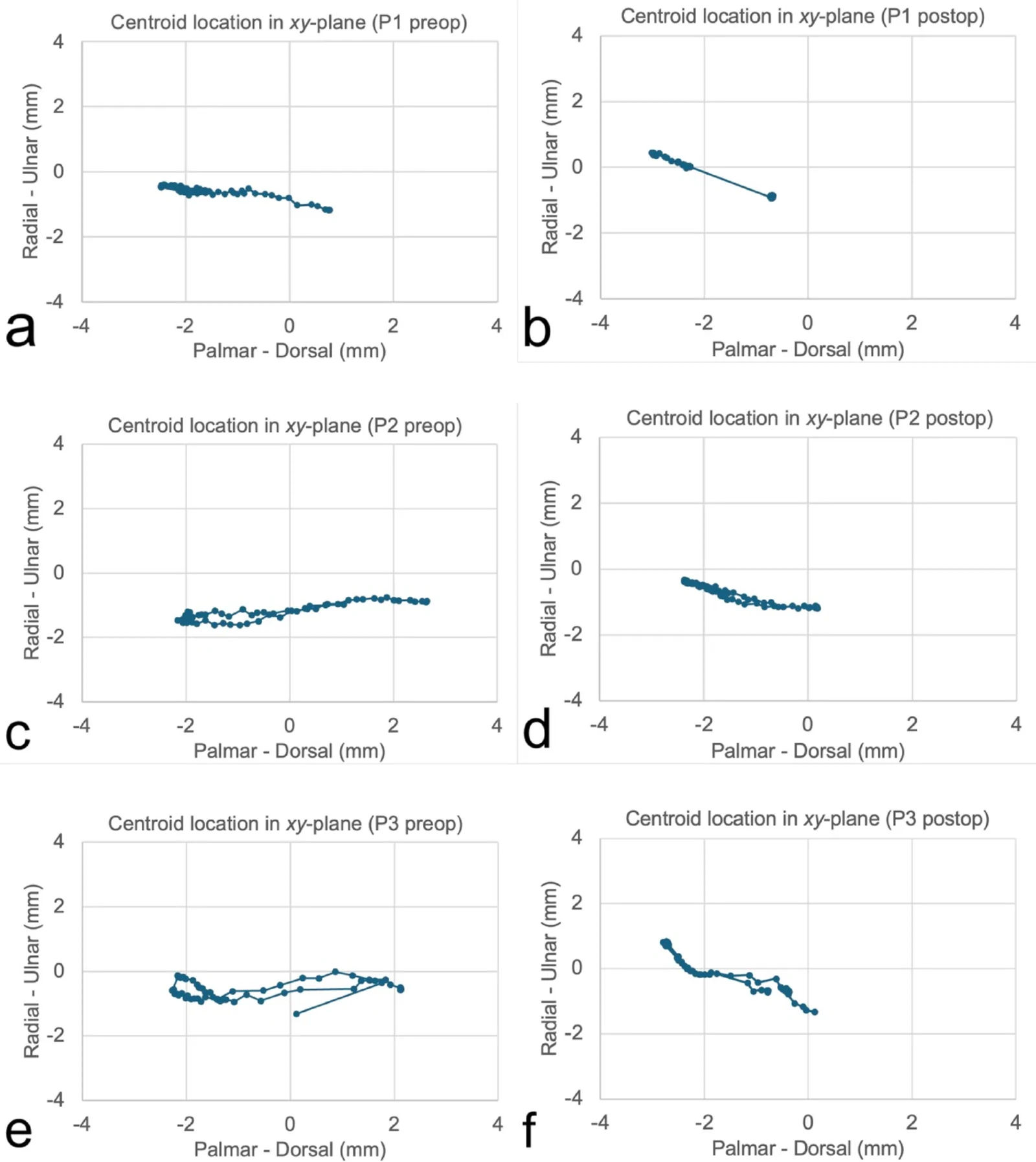

**Figure:**
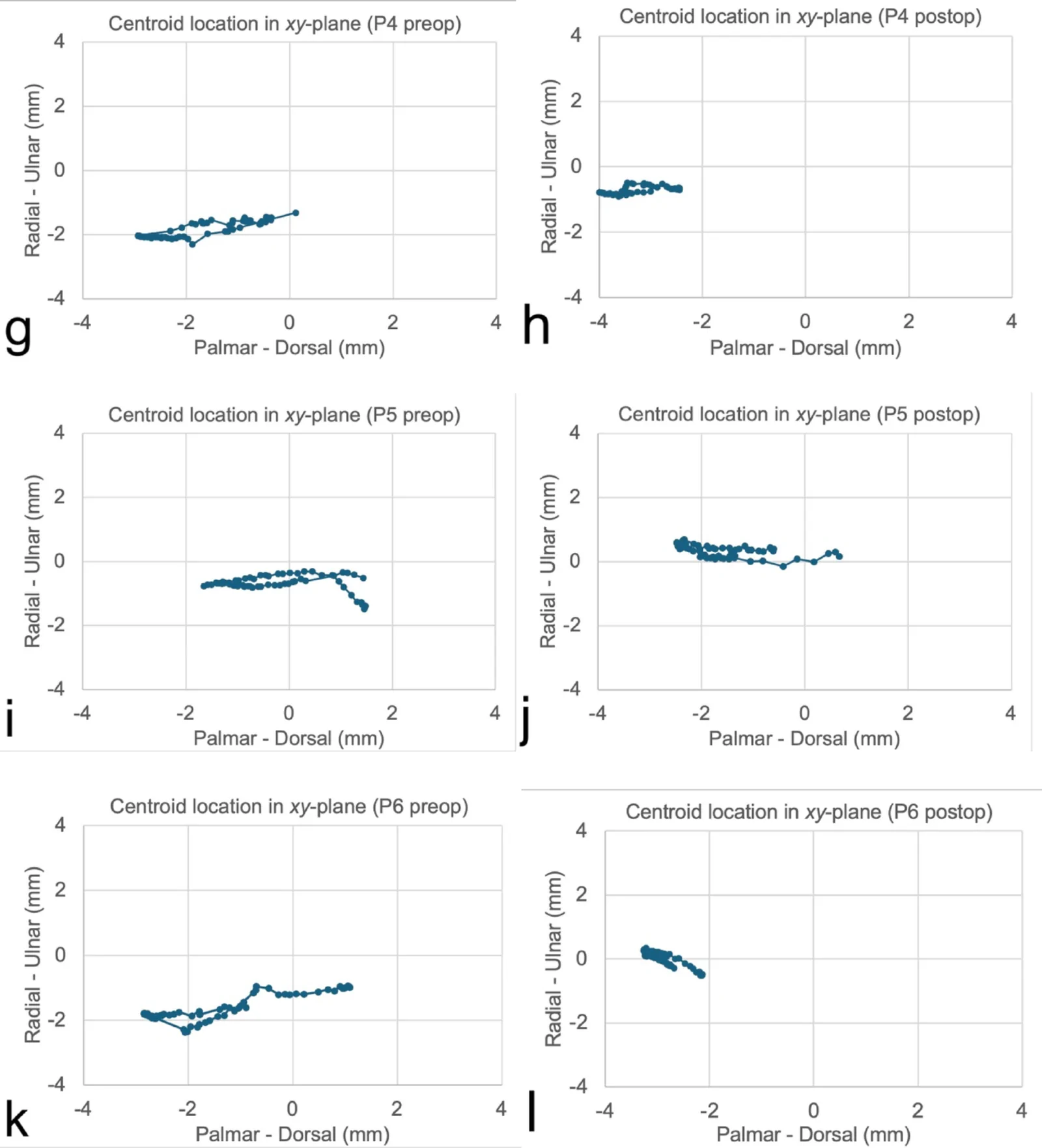


**Table 4:** Centroid path length (mm) in patients (P1-P6) and healthy volunteers (H1-H5)

Patient group			Healthy group	
	Preop	Postop		
P1	5.6	(*)	H1	8.1
P2	9.1	5.6	H2	9.2
P3	12.1	8.4	H3	8.2
P4	7.2	3.3	H4	8.1
P5	7.2	7.7	H5	11.2
P6	7.1	3.5	-	-
Mean	8	5.7		9
SD	2.3	2.3		1.4

The table shows the length of the centroid excursion across the articular surface in mm during opposition – retroposition

(*) Patient did not move during postop opposition 4DCT scan

### Outliers and sensitivity observations

One postoperative dataset (P1) showed a curve shape that did not fit the overall pattern and could not be readily explained from the imaging session or registration logs (Figs. 3a and b and 4 a , b, Tables 2, 3 and 4). The patient stopped moving after opposition during the preoperative scan and started moving abruptly with a delay of approximately 5 s during the postoperative scan. This case was retained for transparency.

## Discussion

This prospective 4D CT study shows that the combined procedure of first metacarpal extension osteotomy and stabilizing ligamentoplasty of the CMC1 joint in early OA does not consistently produce palmar unloading during functional motion. Instead, postoperative mechanics are characterized by a significant increase in palmar contact surface area and shorter centroid path lengths with a net palmar–ulnar shift. Taken together, these changes point toward a stabilizing or stiffening effect on dynamic joint behavior rather than a uniform redistribution from palmar to dorsal surfaces.

Our healthy volunteer data display orderly patterns during opposition and a near-mirror return during retroposition, which accords with prior dynamic CT descriptions of physiologic CMC1 kinematics [14–16, 19]. In contrast, preoperative patient cycles demonstrate loss of mirror symmetry with persistent palmar loading and a more pronounced dorsal-radial bias, which is consistent with symptomatic early osteoarthritis. The postoperative pattern shows increased palmar contact together with shorter centroid trajectories, which differs from the historical expectation derived from pressure film cadaver experiments that emphasized palmar-to-dorsal redistribution under lateral pinch [13]. The divergence is plausible given differences in ligament behavior in vivo, the addition of ligamentoplasty in our cohort, and the fact that our task captured low-load active motion rather than forceful pinch [12, 18].

The study by Nüesch et al. provides an important complementary perspective. Using patient-specific guides and static 3D CT they observed small dorsal–radial shifts of the peak-load centroid and a tendency toward flatter pressure distributions after MC1 osteotomy, although changes were not statistically significant in their limited cohort [22]. Our dynamic data confirm that the combined procedure can reduce focal overloading, but we extend these observations to the temporal dimension: during active motion, we detect robust dorsal–radial unloading and increase in palmar contact area across the cycle together with reduced temporal variability and shorter centroid paths. Differences in imaging modality (dynamic 4D CT versus static 3D CT), task (active motion versus posture), and contact definitions (our 2.0 mm proximity surrogate versus 1.5/0.5 mm thresholds) likely account for the distinct directions and magnitudes of centroid shifts (palmar–ulnar in our series versus subtle dorsal–radial in the series of Nüesch et al.) [22]. These methodological distinctions are clinically relevant because temporal damping and trajectory shortening are not visible on static imaging yet may be responsible for symptom relief.

The automated 4D CT framework reported by Van Royen et al. in healthy volunteers further supports the sensitivity and scalability of motion-resolved imaging [19]. Their pipeline quantified Cardan-angle kinematics, hysteresis, and a proximity-based contact model during a retroposition–opposition–retroposition task. In the present work, we build directly on this paradigm by adding pre- and postoperative patient imaging and by resolving regional contact behavior with quadrant-level metrics and centroid trajectories. The healthy cycles in this study recapitulate the orderly patterns and hysteresis-like return described by Van Royen, whereas the preoperative loss of symmetry and the postoperative increase in palmar contact and shorter centroid excursion provide lacking information on the effect of surgery that their healthy cohort could not address [19].

Clinical series spanning decades, together with a recent systematic review, document reliable pain relief and functional improvement after extension osteotomy, even as strength gains are variable and radiographic progression may continue [3–8]. These outcomes are compatible with the stabilizing mechanism suggested by our imaging, in which damping of dynamic contact excursion rather than palmar unloading explains symptomatic benefit. Contemporary refinements—in particular patient-specific instrumentation and 3D axis analyses—improve correction fidelity and may enhance the consistency of mechanical effects without necessarily altering the underlying mechanism we observed [9–11].

Several limitations deserve emphasis. This investigation was conceived as an observational feasibility study; consequently, no formal a priori power analysis was undertaken. The modest sample size inevitably limits statistical power, particularly for the independent-group comparisons and for the analysis of centroid path length, so that non-significant or borderline findings must be interpreted with caution and may reflect insufficient sensitivity. Nonetheless, the paired analysis demonstrated a statistically significant increase in palmar contact surface area after Wilson osteotomy and modified Brunelli ligamentoplasty, accompanied by a very large effect. These findings indicate that the study was sensitive enough to detect a large within-patient change, while underscoring that the results remain preliminary and require confirmation in adequately powered prospective series. Another relevant limitation is the concomitant stabilization procedure (Brunelli ligamentoplasty) performed with the Wilson osteotomy; thus, it is not sure which intervention attributed which effect on the kinematics of the joint. Furthermore, the thumb mobilization task was performed under low external load, whereas dynamic pinch produces load-dependent kinematics that warrant standardized force control in future work [18]. All proximity-based approaches, including the one described in this study and those used by Nüesch and by Van Royen, model contact as bone-to-bone nearness and therefore cannot directly measure cartilage thickness or contact pressure; the choice of thresholds and regionalization schemes influences apparent contact location and magnitude [19, 20, 22].

In summary, the combined procedure of first metacarpal extension osteotomy and stabilizing ligamentoplasty produced a significant increase in palmar contact surface area, a trend toward shorter contact-centroid paths, and reduced temporal variability during functional motion. Considered together with the patient-specific instrumentation study by Nüesch et al. and the automated 4D CT framework of Van Royen et al., these findings support a predominant mechanism of stabilization and underscore the value of dynamic, region-specific contact analyses for surgical decision-making.

## Supplementary Information

Below is the link to the electronic supplementary material.


Supplementary Material 1



Supplementary Material 2



Supplementary Material 3


## Data Availability

The data that support the findings of this study are available from the corresponding author upon reasonable request, but restrictions apply to the availability of raw imaging data, which were used under licence for the current study and are not publicly accessible.
